# Subtype Distribution of *Blastocystis* spp. in Patients with Gastrointestinal Symptoms in Northern Spain

**DOI:** 10.3390/microorganisms12061084

**Published:** 2024-05-27

**Authors:** Cristina Matovelle, Joaquín Quílez, María Teresa Tejedor, Antonio Beltrán, Patricia Chueca, Luis Vicente Monteagudo

**Affiliations:** 1 Faculty of Medicine, University of Zaragoza, 50009 Zaragoza, Spain; crismatovelle@gmail.com; 2 Environmental Sciences Institute (IUCA), University of Zaragoza, 50009 Zaragoza, Spain; abeltranros@salud.aragon.es; 3 AgriFood Institute of Aragon (IA2), 50013 Zaragoza, Spain; jquilez@unizar.es; 4 Department of Animal Pathology, Faculty of Veterinary Sciences, University of Zaragoza, 50013 Zaragoza, Spain; 5 Department of Anatomy, Embryology and Animal Genetics, Faculty of Veterinary Sciences, University of Zaragoza, 50013 Zaragoza, Spain; ttejedor@unizar.es (M.T.T.); pachueca@unizar.es (P.C.); 6 Aragon Institute of Health Sciences (IACS), Centro de Investigación Biomédica en Red-Enfermedades Cardiovasculares (CIBERCV), 50009 Zaragoza, Spain; 7 Service of Microbiology and Parasitology, Hospital Clínico Universitario Lozano Blesa, 50009 Zaragoza, Spain

**Keywords:** *Blastocystis* spp., genetic diversity, phylogenetic analysis, subtypes

## Abstract

Limited molecular data exist on the prevalence and subtype distribution of *Blastocystis* spp., the most prevalent parasite in human and animal feces worldwide. A total of 44 different subtypes (STs) of *Blastocystis* are currently recognized based on the sequence of the small subunit ribosomal RNA (*SSU-rRNA*) gene. This is a molecular study of *Blastocystis* spp. in hospitalized patients with gastrointestinal symptoms in northern Spain. We analyzed 173 *Blastocystis*-positive patients with gastrointestinal symptoms by using nested PCR for molecular detection, subtype identification, phylogenetic analyses, and genetic diversity assessment. ST2 (34.1%) and ST3 (34.7%) predominated, followed by ST1 (15.6%) and ST4 (15.6%). Mixed infections with different subtypes were observed in some patients. Sequence analysis revealed for the first time in European humans the allele 88 (a variant of ST1). In other cases, alleles commonly found in animal samples were detected (allele 9 in ST2, allele 34 in ST3, and allele 42 in ST4). Phylogenetic analysis showed high variability in ST1 and ST2, suggesting a polyphyletic origin, while both ST3 and ST4 exhibited higher genetic homogeneity, indicating a possible monophyletic origin and recent transmission to humans. These data confirm *Blastocystis* spp. subtype diversity and may help in understanding the evolutionary processes and potential zoonotic transmission of this parasite.

## 1. Introduction

*Blastocystis* spp. is the most prevalent intestinal protozoon detected in humans and animals worldwide. This enteric protist is known to be associated with gastrointestinal symptoms in people across both industrialized and developing countries [1]. Various factors account for the high prevalence of *Blastocystis* spp. Its transmission occurs through the fecal–oral route, with several sources of infection, including person-to-person, zoonotic, and waterborne transmission. In developing countries, it is associated with socio-economic factors leading to poor sanitation [2]. This protozoon could have implications for public health since it can be transmitted to humans from animals, suggesting its potential zoonotic nature. The “One Health” strategy, promoted by the World Health Organization, encourages interdisciplinary collaboration to achieve optimal health for humans, animals, and the environment, effectively addressing zoonotic infections like *Blastocystis* spp. This approach enhances the understanding of the disease and facilitates the adoption of specific control measures for the benefit of both human and animal populations [3]. 

Over the past decade, the scientific community has increasingly focused its attention on unraveling the genetic diversity of *Blastocystis* spp. by using the small subunit ribosomal RNA (*SSU-rRNA*) gene as a molecular marker. This genetic tool has yielded invaluable insights into its taxonomy, population structure and potential pathogenicity, significantly enhancing our comprehension of *Blastocystis* spp. [4]. A total of 44 different subtypes and numerous subtype subgroups have been reported to date based on variations in the *SSU-rRNA* gene in humans and animals [5]. However, not all strains of a specific subtype have confirmed clinical significance and the potential relationship between different subtypes and their ability to cause disease is still a topic of active debate [6].

Infection with *Blastocystis* spp. in humans has been reported across the globe [7,8]; in Europe, the reported prevalence of this protist in humans ranges from 3% to 7% in France, Italy and the United Kingdom (UK) by using optical microscopy, but higher prevalence levels (14.5–24.2%) were found when PCR-based studies were conducted in France, the Netherlands and Denmark [9,10,11,12,13,14]. Subtype ST3 exhibits the highest global distribution, with subtypes ST1 and ST2 following closely in prevalence [15]. In Europe, subtype ST3 is also the most frequent [16,17,18], followed by ST4 [16,19], then ST2 [20,21] and lastly ST1 [11,22]. Additional *Blastocystis* subtypes that are rare in the human population have been identified in Europe, such as ST5 [18], ST6 [23], ST7 [24], ST8 [21], and ST9 [25]. 

A long-debated topic is related to the pathogenicity of *Blastocystis* spp. and the ongoing challenge to determine whether this protozoon is genuinely pathogenic, a commensal or only pathogenic in specific situations such as immunosuppression, malnutrition or recurrent infections [26,27]. Some studies suggest that *Blastocystis* spp. could be part of a healthy intestinal microbiota, potentially mitigating inflammation and autoimmune disorders; it prompts interleukin-22 release, thus assisting in intestinal mucosal secretion to relieve colitis symptoms and it may also contribute to host metabolism by breaking down cellulose [28]. Researchers have also explored pathogenicity variations among *Blastocystis* subtypes (ST), yet conclusive findings remain elusive. ST1, ST2, and ST4 are implicated as potential sources of gastrointestinal symptoms, with studies indicating their higher prevalence in symptomatic patients compared to controls. ST1 has been linked to irritable bowel syndrome [29], while ST2 is associated with gastrointestinal issues and urticaria, and is particularly prevalent in patients with diarrhea in Colombia, whereas asymptomatic individuals carry ST1 [30]. However, some studies present inconsistent support for ST2’s pathogenicity [31]. ST3 is predominantly found in patients with urticaria and gastrointestinal symptoms [32] while ST4 shows high prevalence in severe diarrhea cases [33]. Subtypes ST5, ST6 and ST7 also exhibit potential pathogenicity [34]. Although rare in humans, ST8 has been linked to severe symptoms in two studies [35].

In Spain, the reported prevalence of *Blastocystis* in human populations is highly variable, and it could be grossly underestimated in several studies due to the low diagnostic sensitivity of some detection techniques; specifically, molecular analyses are much more sensitive than microscopy and in vitro xenic culture for detecting *Blastocystis* spp. in fecal samples from humans and animals [19,36]. A recent study conducted in Zaragoza (Spain) using conventional microscopy found a prevalence of 9.2% [37]. This result is consistent with other research using conventional microscopy in the central region of Spain, where a prevalence value of 9.6% was documented in HIV-positive children, and 5.3–19.4% in children attending nurseries and primary schools [38,39]. Nevertheless, the figure rises to 13% among asymptomatic schoolchildren using PCR-based methods in the same geographical area of central Spain [40]. Higher values (27.8%) have been reported in adult patients in northeastern Spain using microscopic examination and PCR [41], while a similar procedure reported a prevalence of 35.2% among humans cohabiting with dogs and cats in northern Spain [42]. Studies investigating the subtype distribution of *Blastocystis* spp. are limited in Spain and predominantly focused on specific population groups. Subtypes ST1–ST4 and ST8 have been identified in both asymptomatic and symptomatic schoolchildren in Madrid. Subtype ST4 was the most prevalent in a human population in Valencia, while subtype ST2 was the most frequently detected in Alava [20,21,33,37,42]. The aim of the current study was to analyze the genetic diversity of *Blastocystis* spp. subtypes circulating in infected patients in an area of northern Spain and to investigate any differences in clinical significance among the various subtypes.

## 2. Materials and Methods

### 2.1. Ethics Approval Statement

This study was conducted in accordance with the guidelines of the Declaration of Helsinki (1975, revised in 2013) to ensure ethical considerations in human research. Approval for this study was obtained from the Ethics Committee of Aragón (ref 18/081) before commencing the research, ensuring compliance with national and international guidelines. All participating patients were anonymized and provided signed informed consent. This study also adheres to the requirements of the Health Insurance Portability and Accountability Act (HIPAA, 1996). Throughout the research, mandatory health and safety procedures were adhered to.

### 2.2. Sampling of Fecal Specimens

A total of 6807 stool samples from 3682 patients showing gastrointestinal symptoms in the year 2018 were analyzed as described in a previous report [37]. Among the 338 *Blastocystis*-positive (by microscopy) patients detected in that previous report, 173 fecal samples providing good DNA sequences (following the procedure described in the next sections) were included in the present study. *Blastocystis* positivity and sufficient quality of the genetic sequence were the inclusion criteria. The categorical variables analyzed for association with *Blastocystis* spp. infection were: demographic origin (Spain, rest of Europe, Africa, American continent and Asia); age group (16 years or younger and >16 years); gender (male and female) and *Blastocystis* subtypes (ST1, ST2, ST3 and ST4 subtypes). 

### 2.3. Molecular Detection of Blastocystis spp.

According to the manufacturer’s instructions, DNA extraction was performed on the 173 samples using a DNA Stool Kit (NORGEN BIOTEK CORP., Thorold, ON, Canada). For the molecular detection of *Blastocystis*, a nested PCR was performed. The primary PCR amplified the conserved eukaryotic region of the 18S rRNA gene with universal primers EUK-F and EUK-R in a 50 μL final volume [43]. For the secondary PCR, a specific *SSU-rRNA* gene fragment of *Blastocystis* spp. was amplified following the protocol by Santín et al. [44] using the primary PCR product as a template. The primers Blast 505–532 and Blast 998–1017 amplify a ~479 bp fragment, including a variable region of the *SSU-rRNA* gene that enables the subtyping of *Blastocystis* spp. The reaction mix for the secondary PCR was prepared in a final volume of 50 μL. Both PCR reactions were performed using a MJ Research MINICYCLER-PCR-THERMAL CYCLER and Applied Biosystems™ 2720 Thermal Cycler (Applied Biosystems, Whaltman, MA, USA). In order to verify that the PCR generated amplicons were of the desired size, agarose gel electrophoresis was performed using the products from the secondary PCR.

### 2.4. Subtype Identification, Phylogenetic Analyses and Genetic Diversity

The PCR products were purified using the Speedtools PCR Clean Up Kit (Biotools, Madrid, Spain) and sequenced on both strands by the Sanger method. The sequences obtained were edited and assembled in BioEdit software version 7.0.0 URL https://bioedit.software.informer.com/7.0/ (accessed on 24 May 2024). To confirm the identity of the sequences as *Blastocystis* spp., they were compared with the reference sequences of the different *Blastocystis* spp. subtypes available in the GenBank^®^ database using the nucleotide BLAST program provided by the National Center for Biotechnology Information (NCBI) [45,46]. Subsequently, the sequences were assembled in FASTA format and submitted to the *Blastocystis* Subtype database (18S), which is a multilocus sequence typing (MLST) database available at http://pubmlst.org/blastocystis/ (accessed on 24 May 2024), and the ST and corresponding alleles were determined through sequence comparison. As of 15 January 2024, the database contained 357 alleles for the gene investigated in the present study [47,48]. 

Following alignment using ClustalW [49] in BioEdit 7.0 [50], the *SSU-rRNA* gene sequences of *Blastocystis* spp. were analyzed. Finally, phylogenetic analysis was performed using the Neighbor Joining (NJ) method based on genetic distances calculated using the 2-parameter or Kimura 2 model [51] with MEGA5.10 software [52]. A sequence from *Proteromonas lacertae* (GenBank^®^ accession number U37108) was used as an outgroup. The resulting trees were exported in Newick format (which allows tree representation using parentheses and commas) [53]. For the graphical representation of the obtained phylogenetic trees, the online software iTOL v5 (https://itol.embl.de/about.cgi, accessed on 24 May 2024) [54] was used. 

To assess the genetic diversity of the sequences, the DNAsp v6.12.01 software [55] available at http://www.ub.edu/dnasp/ (last accessed on 15 January 2024) was used. The parameters used to measure genetic diversity among the sequences included the number of polymorphic sites (S) [a site is considered polymorphic when different sequences yield distinct nucleotides at that site or position], number of haplotypes (h) [each specific combination of nucleotides in the sequence is considered a different haplotype], haplotype diversity (Hd) [the likelihood of two randomly sampled haplotypes being distinct], and nucleotide diversity (π) [the average number of nucleotide differences per site between two sequences]. 

## 3. Results

### 3.1. Subtypes

A total of four different subtypes were identified among the 173 *Blastocystis*-positive samples that were previously analyzed in the GenBank^®^ database. The predominant subtypes were *Blastocystis* ST2 (34.1%) and ST3 (34.7%), which were found in a similar proportion of patients, followed by subtypes ST1 (15.6%) and ST4 (15.6%), which were both identified in an equal number of infected patients. Only 111 among the 173 *Blastocystis*-positive samples met the requirements of length and quality to be deposited in the GenBank^®^ database with the accession numbers OP495227–OP495337 and to be compared with sequences previously deposited in the database by means of BLASTn [45,46]. Nevertheless, after aligning the *Blastocystis* spp. sequences and identifying a partial length bias in some sequences, the shortest ones were discarded, leaving only 83 sequences that met the length requirements (minimum 352 bp) to be selected for genetic diversity study and phylogenetic analysis. Genetic diversity analysis performed in the 352 aligned nucleotide sites provided nucleotide diversity per site (Pi = 0.14808) and an average of 50.05260 nucleotide differences between haplotypes. 

Out of the 173 *Blastocystis* samples, 11 (6.3%) exhibited mixed *Blastocystis* sequences, which were identified by double peaks in the *SSU-rRNA* gene chromatograms. These peaks occurred within a 30-nucleotide segment, enabling specific variant identification within the rest of the sequence. Four out of these eleven sequences belonged to the subtype ST1 (36.4%) and seven to the subtype ST2 (63.6%). Another significant finding was the subtype variation observed in four of the patients who submitted repeated samples. Namely, one patient switched from ST1 to ST3 in nine days, another from ST3 to ST1 in just one day. Notably, one patient showed ST1, then ST2 after three days, and reverted to ST1 three days later (Table 1).

### 3.2. Alleles

Analysis of allele distribution according to the age, gender and geographic origin of the patients is indicated in Tables S1–S4. Allele 88 was the most common within ST1-positive patients (9/12; 75%) followed by alleles 2 (2/12: 16.6%) and 4 (1/12, 8.33%); allele 2 was found in both Spanish and European patients. ST2 sequences featured only two alleles: allele 9 (15/23; 65.2%) and allele 13 (8/23; 34.8%), with patients from different geographic distributions (Table S2). ST3 sequences were mainly associated with allele 34 (29/32; 90.6%), while three samples showed allele 36 (3/32, 9.4%), and most of them (31/32; 97%) originating from patients born in Europe and Africa (Table S3). The 16 ST4 sequences uniformly exhibited allele 42, with all patients being of Spanish origin, and a majority (10/16; 62.5%) being under 16 years of age (Table S4). 

### 3.3. Blastn Alignments with Sequences Existing in GenBank

The 111 *Blastocystis* spp. sequences were subjected to BLASTn comparisons with reference sequences deposited in GenBank [45,46] (Tables S5–S8). Notably, some ST1 and ST4 sequences showed 100% similarity with *Blastocystis* sequences from animals (*Bos taurus*, *Sus scrofa domesticus*) but lower similarity with strains from humans, suggesting a potential zoonotic origin. In contrast, most ST2 and ST3 sequences displayed the highest similarity with human-origin sequences, suggesting a potential human-to-human transmission pattern.

### 3.4. Genetic Diversity and Phylogenetic Analysis

Nucleotide comparisons among partial *SSU-rRNA* gene sequences of each *Blastocystis* subtype are shown in Tables S9–S12. Comparison of the ST1 subtype revealed that all 17 sequences had a nucleotide similarity higher than 94.3% to each other but only eight sequences were 100% identical. The number of sequences exhibiting 100% identity to each other was greater within subtypes 2 and 3. Notably, all but two of the twenty sequences belonging to the ST4 subtype showed 100% similarity to each other. 

Among 83 *Blastocystis* spp. sequences that met the requirements to be selected for genetic diversity study and phylogenetic analysis, 26 distinct haplotypes were identified, yielding a joint haplotype diversity index of 0.927. Genetic diversity was assessed for each *Blastocystis* spp. subtype (Table 2). ST1, ST2, ST3, and ST4 displayed eight, seven, nine and two haplotypes, respectively. Notably, ST1 exhibited the highest haplotype diversity (Hd: 0.924), while ST4 had the lowest (Hd: 0.233), with 30 and 1 polymorphic sites, respectively. ST2 showed the highest nucleotide diversity (π: 0.03945), indicating substantial variability in the *SSU-rRNA* gene fragment. 

**Figure 1:**
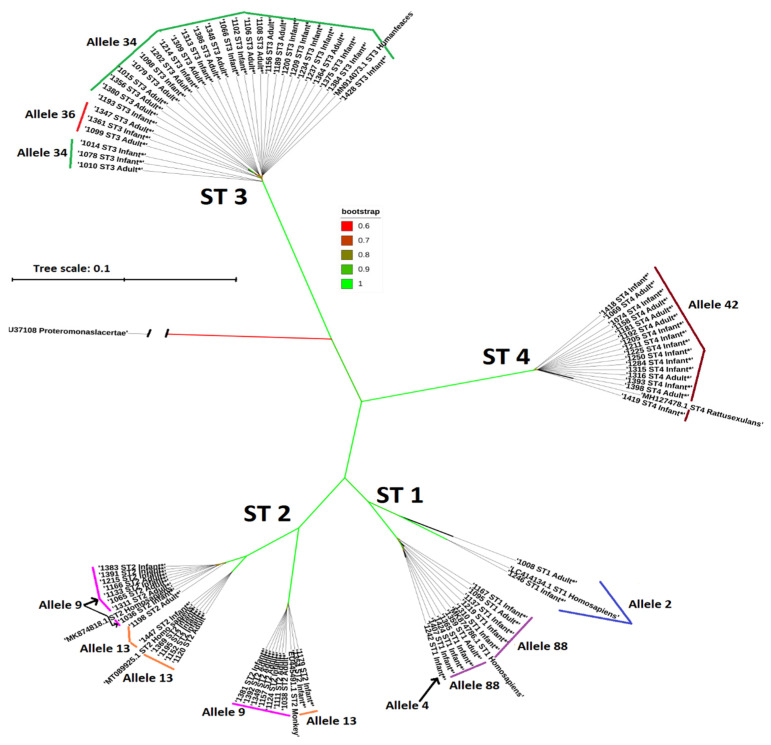
Neighbor joining analysis of the partial sequences of the *SSU-rRNA* gene of *Blastocystis* and reference sequences representative of different subtypes. A sequence of *Proteromonas lacertae* was used as the outgroup and sequences of other *Blastocystis* subtypes were obtained from GenBank^®^ (LC414134.1 and MK874786.1: ST1 *Homo sapiens*, EU445491.1: ST2 Monkey (sic.), M25.1 and MK874818.1: ST2 *Homo sapiens*, MN914073.1: ST3 *Homo sapiens*, and MH127478.1: ST4 *Rattus exulans*). Genetic distances were calculated using the Kimura 2 model (own image). The length of the branch connecting the outgroup sample was reduced by 61% to simplify the image. The symbol * indicates the sequences obtained in the present work.

Figure 1 presents a phylogenetic tree of the *SSU-rRNA* gene sequences of *Blastocystis* rooted with *Proteromonas lacertae* (U37108) as the outgroup. Four well-supported branches (green) with 1000 bootstrap replicates (support value of one) are depicted. In the ST1 branch, observed in the vertical representation, two well-supported sub-branches with an absolute support value of one are apparent. The first sub-branch holds two sequences associated with allele 2, while the second sub-branch comprises nine sequences displaying allele 88, along with one sequence with allele 4. In ST2, three well-supported sub-branches emerge from the bootstrap replicates. The first branch includes seven sequences linked to allele 9 and two to allele 13. The second group comprises solely allele 13 sequences, while the third contains allele 9 sequences, except for one linked to allele 13. Within the ST3 branch, two distinct alleles are observed: allele 34 dominates most sequences, while only three sequences correspond to allele 36. On the opposite side, in the ST4 branch, all sequences exhibit allele 42, and no sub-branches are observed, indicating a possible recent monophyletic pattern.

## 4. Discussion

The present report is based on the analysis of fecal samples obtained from patients exhibiting gastrointestinal symptoms in a limited geographical area of Spain (not from healthy individuals). In spite of the limited sample size, marked genetic variability was observed in the set of samples. The distribution of *Blastocystis* spp. subtypes varies across countries and continents, as summarized in Tables S13 and S14 [56]. The laboratory protocol used in the present study, based on PCR amplification of a 479 bp fragment of the *Blastocystis SSU-rRNA* gene and proposed by Santín et al. [44], is widely utilized in specialized literature, even if other methodologies are used too. 

The current study identified ST3 and ST2 as the most prevalent subtypes. Subtype ST3 is the predominant *Blastocystis* subtype globally [23,57,58,59,60]. In Spain, this subtype was not detected in symptomatic individuals in Valencia but ranked second among pets and their owners in Álava and was less common in the Madrid region [20,21,33,42]. 

In warm climates like those in Senegal, Lebanon, Saudi Arabia and Bolivia, ST2 is among the most common subtypes, suggesting adaptation to such environments, even if it was also the most prevalent one in a study performed in Ireland [9,13,23,24,59,60,61]. The high frequency of ST2 and ST3 in South America has been associated with poverty, sanitation issues, civil conflicts, biodiversity and limited access to clean water, promoting *Blastocystis* spp. transmission [8]. ST2 was also the most frequent subtype reported in previous Spanish studies in Alava, Leganés (Madrid) and Central Spain, being mostly associated with children, even when they were asymptomatic [20,21,42]. Conversely, ST2 was less common in Valencia, where most fecal specimens were from adult patients [33]. These observations suggest a potential fecal–oral transmission cycle among school-age children for ST2 [62]. 

In our study, both ST4 and ST1 were the less-prevalent *Blastocystis* subtypes, each detected in over 15% of patients. The ST4 subtype is commonly found in temperate European countries like Denmark and France, while it is infrequent in Japan, Malaysia, China, Latin American and African countries [1,16,58,60,63,64,65,66]. In Spain, ST4 was previously found to be highly prevalent in Valencia (over 94% of *Blastocystis*-infected patients) [33]. ST1 is among the less common subtypes in Europe, except in some studies in Germany, Greece, France, Denmark and Italy [22,24,61,67,68]. Conversely, ST1 is the most common *Blastocystis* variant in several countries across the rest of the continents, including Colombia, Brazil, Libya, Nigeria, Tanzania, Iran, Turkey, Saudi Arabia, the Philippines and Australia [18,44,69,70,71,72,73]. 

A high proportion (42%) of the patients studied in the present report are immigrant residents from various countries [37]. This could be a possible explanation for the different *Blastocystis* subtype distribution when compared with that found in other points of Spain, highlighting the need for molecular studies in order to understand *Blastocystis* spp. genetic diversity globally.

A search in GenBank^®^ via Blastn revealed that some of the sequences obtained in this research exhibit 100% identity with previous isolates obtained from animals and human beings all over the world. For instance, the ST1 sequences closely match those from humans in several countries such as Colombia, Mexico, Laos and Malaysia [44,74]. Such coincidences among samples from distant origins are not uncommon: in a Chinese study, two ST1 sequences were identical with a *Blastocystis* sequence from humans in Turkey [75]. 

Our ST2 sequences matched those from human samples globally and from various animal species in Germany, Spain, China and Iran [9,13,44,74,76,77,78,79]. 

The ST3 sequences showed similarities with sequences from human samples from Mexico, Colombia, Germany, South Korea, the Philippines, Malaysia, Libya, Egypt and Turkey, as well as high similarity with animal sequences from the USA, Spain, Iran, Malaysia, China and Japan [44,80,81,82,83,84]. This observation is consistent across multiple studies owing to the widespread prevalence of ST3 globally [44,77,81,85,86,87]. 

The ST4 sequences also showed high similarity with sequences of human and animal origin from various regions [77,88,89,90]. Numerous ST4 sequences in GenBank^®^ are linked to various rodent species, supporting their role as primary hosts for this subtype [91,92,93,94]. 

In Spain, diverse investigations explored *Blastocystis* spp. isolates in animals and the environment. A study on fecal samples from free-living carnivores in various regions identified ST2 and ST4 *Blastocystis* subtypes in red fox, exhibiting significant sequence similarity (98.5–100%) to those in the present study [95]. Similar patterns were observed in urban wastewater in Valencia, where ST2 was the predominant subtype [96]. Additionally, 100% identity was found with ST3 sequences from *Blastocystis* isolates from cattle from Álava [83] and a 99.5% similarity was found with ST4 sequences from *Blastocystis* in *Rattus* spp. from a zoo in Córdoba [97]. The substantial similarity among *Blastocystis* spp. isolates across different regions and animals implies a potential zoonotic connection, raising public health concerns. Widespread international trade and travel could explain the identical sequences observed between our study and those from distant countries.

This study found mixed *Blastocystis* spp. infections with diverse subtypes, in agreement with worldwide observations. In contrast to previous Spanish studies, a French investigation utilized PCR product cloning to identify three subtypes (ST2, ST3, and ST4) within the same host [20,21,33,42,98]. However, the practical use of this technique in larger cohorts is hindered by its labor-intensive nature and its high cost. Various protocols, including the one in this study, have detected mixed infections using DNA from in vitro cultures or fecal samples. Sanger sequencing of PCR-amplified 18S rRNA gene products, with universal or *Blastocystis*-specific primers, has been used widely. Overlapping peaks in chromatograms indicate mixed infections, with PCR often amplifying DNA from the predominant subtype. In cases of similar subtype concentrations, the amplifications of both of them are equally efficient and result in double peaks [16,77,99]. Next-Generation Sequencing (NGS) has revolutionized the detection of mixed *Blastocystis* spp. infections, providing precise quantification even at low levels (as low as 5%). However, its application is restricted by high costs and the need for highly skilled personnel [84]. Mixed *Blastocystis* infections are frequently overlooked in research due to detection limitations. In a meta-analysis, fewer than half of the studies (24/55) reported mixed *Blastocystis* infections, with a prevalence of around 6% [18,100]. Our study mirrors this prevalence, identifying mixed infections in 6.3% (11 out of 173) of subtyped samples. Four of these eleven samples belonged to ST1 and seven to ST2.

The finding of different subtypes in repeated samples of the same patient is also an unexpected observation in this study. PCR amplifies the predominant subtype, raising uncertainty about whether undetected coexisting subtypes were present initially due to technical limitations. In one case involving three samples, the detected subtype in the first and last samples was the same. Another explanation could be reinfection with a different strain, but this is less plausible because of the short interval between the two samples (1–9 days, depending on the patient). It is noteworthy that three of the four individuals showing this pattern were infected with ST1, which is a minor subtype in this study but commonly found in mixed infections [101].

The DnaSP results indicate varying levels of diversity, with ST1 sequences exhibiting the highest number of haplotypes and polymorphic sites and ST4 sequences showing the lowest diversity. In a study in Iran, ST2 had the highest haplotype diversity (Hd: 0.934) and ST1 the lowest one (Hd: 0.564) [102]. Conversely, ST1 was the most variable subtype in a study in Saudi Arabia [71]. In our study, ST2 also showed the highest nucleotide diversity (π: 0.03945). Overall, these findings confirm significant variability in the amplified *SSU-rRNA* gene fragment among *Blastocystis* subtypes, suggesting a longer evolutionary history for ST1 and ST2, while ST3 and ST4 are more recent. In fact, the low variability observed in ST4 could also be due to a recent transmission of this subtype to humans. The sequences for ST4 obtained in rats, Guinea pigs and opossum and most human patients show a high similarity. Rodents are considered a possible reservoir for *Blastocystis* transmission to humans [30,103]. 

Various authors highlight the value of distinguishing *Blastocystis* alleles for insights into host specificity, geographic distribution and clinical manifestations [30,104,105]. In our study, allele diversity within each subtype was limited. ST1 exhibited the highest diversity with three identified alleles (2, 4, and 88), consistent with findings by other authors. While allele 4 is commonly reported in European studies, allele 88, previously identified in the Middle East and South America, especially in immunocompromised patients, has been detected for the first time in European human populations in the present report [106,107,108].

Two alleles (9 and 13) were identified within ST2 sequences, with 9 being the most frequent. Previous research [1,30,105] revealed that allele 9 is the most prevalent in humans and animals in South America, too [20]. In contrast, other studies identified allele 12, which was not observed in this study, as the most common in humans and animals, and did not find allele 9 [21,42,97]. Among the fifteen sequences with allele 9 in our study, over a third (7/15) were from individuals from Africa (five) and from South America (two). Furthermore, allele 9 has been observed in studies involving dogs and rats, indicating potential zoonotic transmission [1,109]. 

In ST3, we identified two alleles (34 and 36), with a clear predominance of the former (29/32 ST3 isolates), consistent with the higher frequency of allele 34 in the European human population [21,42,97]. In contrast, allele 36 is dominant in African children, where some studies do not detect allele [15,34,110]. Sequences of ST4 exhibited minimal variability in our study, with only allele 42 being observed, consistent with other research [21,110]. ST4 is prevalent in rodents and the previous detection of allele 42 in stray cats suggests their potential role as a source of *Blastocystis* spp. infections in humans [111]. 

Neighbor joining analysis showed more than one branch in the phylogenetic tree for both ST1 and ST2 sequences. This suggests possible diverse sources of infection from humans or animals with these subtypes and potential ancient origins and extensive evolutionary processes [112]. In contrast, the dominant ST3 exhibited a limited variability, which could be explained by a higher infectivity, by a potential origin from limited sources or by a shorter evolutionary timeline. The greatest genetic homogeneity was observed in ST4 sequences, suggesting that its transmission to humans is more recent than that of the other subtypes.

Furthermore, it is noticeable that ST4 sequences from rats, guinea pigs, opossums and most humans are highly conserved. This supports the theory that rodents can be a reservoir for human infections with *Blastocystis* ST4, as suggested in other works [30,103]. In the present dataset, patients showing this subtype were of Spanish origin; the only two sequences presenting similarity slightly below 100% came from patients living in a rural area.

## 5. Conclusions

In summary, this molecular study revealed that the distribution of *Blastocystis* spp. subtypes infecting humans showed variations compared to other geographical areas in Spain, a circumstance that could be related to the high percentage of immigrants residing in the population investigated. Two predominant subtypes (ST2 and ST3) and two minor subtypes (ST1 and ST4) have been identified. Also, in some patients, mixed infections with different subtypes of *Blastocystis* spp. have been detected by the presence of double peaks in chromatograms following Sanger sequencing of the *SSU-rRNA* gene. The analysis of the sequences of the different subtypes has revealed an allele previously undescribed in European human samples (allele 88 in ST1) and other alleles coincident with those found in animal samples (9 in ST2, 34 in ST3 and 42 in ST4). In fact, high genetic similarity has been found with isolates of *Blastocystis* spp. from samples obtained in both human and animal species obtained in geographically distant regions; these findings could support the potential zoonotic nature of this parasite. The highest genetic variability was observed in ST1 and ST2, suggesting a polyphyletic origin of these variants, indicative of diverse origins or a longer evolutionary process. The greatest similarity was observed among the ST3 and especially ST4 sequences, indicating a probable monophyletic origin and/or a more recent transmission for these subtypes.

## Figures and Tables

**Table 1:** Patients with variation of subtypes in repeated samples.

Patient	Sample Date	Subtype	Age (years)	Country of Birth
1	17 September 2018	ST1	2	Ukraine
	26 September 2018	ST3		
2	3 October 2018	ST3	70	Spain
	4 October 2018	ST1		
3	9 January 2018	ST1	8	Gambia
	12 January 2018	ST2		
	15 January 2018	ST1		
4	19 February 2018	ST4	62	Spain
	22 February 2018	ST2		

**Table 2:** Genetic variability of *Blastocystis* spp. subtypes in patients in the present study.

Subtypes	Frequency	Monomorphic Sites	Polymorphic Sites	h*	Hd^≠^	π^#^
ST1	12	315	30	8	0.924	0.02429
ST2	23	316	28	7	0.842	0.03945
ST3	31	342	7	9	0.774	0.00438
ST4	16	346	1	2	0.233	0.00067
Total	83	234	104	26	0.927	0.14808

h*: Number of haplotypes, Hd^≠^: Haplotype diversity, π^#^: Nucleotide diversity.

## Data Availability

The sequences used for analysis in the present manuscript are available in the GenBank repository [www.ncbi.nlm.nih.gov/genbank/] [OP495227-OP495337]. The other datasets are available on request from the authors.

## Supplementary Materials

The following supporting information can be downloaded at: https://www.mdpi.com/article/10.3390/microorganisms12061084/s1, Table S1. Distribution of *Blastocystis* sp. alleles, country of origin of the patients, age and sex in sequences belonging to ST1. Table S2. Distribution of *Blastocystis* sp. alleles, country of origin of the patients, age and sex in sequences belonging to ST2. Table S3. Distribution of *Blastocystis* sp. alleles, country of origin of the patients, age and sex in sequences belonging to ST3. Table S4. Distribution of *Blastocystis* sp. alleles, country of origin of the patients, age and sex in sequences belonging to ST4. Table S5. Comparisons of the genetic sequences obtained from ST1 with the genetic sequences stored in the GenBank^®^ database. Table S6. Comparisons of the genetic sequences obtained from ST2 with the genetic sequences stored in the GenBank^®^ database. Table S7. Comparisons of the genetic sequences obtained from ST3 with the genetic sequences stored in the GenBank^®^ database. Table S8. Comparisons of the genetic sequences obtained from ST4 with the genetic sequences stored in the GenBank^®^ database. between partial SSU-rRNA gene sequences identified as *Blastocystis* ST1. There was a total of 136 combinations in the final data set. The pink background indicates the maximum range (100%) and the green background the minimum range (94.26%). Table S9 Homology between partial SSU-rRNA gene sequences identified as *Blastocystis* ST1. There was a total of 136 combinations in the final data set. The pink background indicates the maximum range (100%) and the green background the minimum range (94.26%). Table S10 Homology between partial SSU-rRNA gene sequences identified as ST2 of *Blastocystis*. There was a total of 465 combinations in the final data set. The pink background indicates the maximum range (100%) and the green background the minimum range (94.72%). Table S11. Homology between partial SSU-rRNA gene sequences identified as ST3 of *Blastocystis*. There was a total of 903 combinations in the final data set. The pink background indicates the maximum range (100%) and the green background the minimum range (91.38%). Table S12 Homology between partial SSU-rRNA gene sequences identified as ST4 of *Blastocystis*. There was a total of 190 combinations in the final data set. The pink background indicates the maximum range (100%) and the green background the minimum range (99.72%). Table S13. Distribution of *Blastocystis* sp. subtypes by continents. Table S14. Frequency of subtypes (%) of *Blastocystis* sp. identified in human infections in various studies in Spain.
