# Supplementary material for: Subtype Distribution of Blastocystis spp. in Patients with Gastrointestinal Symptoms in Northern Spain
Source: Microorganisms. 2024 May 27;12(6):1084. doi: 10.3390/microorganisms12061084 (PMC11205523; doi:10.3390/microorganisms12061084)
Supplement: Supplementary file 1 [file microorganisms-12-01084-s001.zip › microorganisms-3027090-supplementary.pdf]

Table S1 Distribution of *Blastocystis* sp. alleles, country of origin of the patients, age and sex in sequences belonging to ST1

Allele	Geographical origin	Age	Sex
2	Spain	> 16 years	Male
88	Rest of Europe	> 16 years	Male
88	Spain	≤ 16 years	Male
88	Africa	≤ 16 years	Female
88	Asia	≤ 16 years	Female
4	Africa	≤ 16 years	Female
2	Rest of Europe	≤ 16 years	Female
88	Africa	≤ 16 years	Male
88	Spain	> 16 years	Male
88	Rest of Europe	≤ 16 years	Female
88	Spain	≤ 16 years	Male
88	Asia	≤ 16 years	Female

Table S2 Distribution of *Blastocystis* sp. alleles, country of origin of the patients, age and sex in sequences belonging to ST2

Allele	Geographical origin	Age	Sex
9	Spain	≤ 16 years	Female
9	Africa	> 16 years	Male
9	America	> 16 years	Male
9	America	> 16 years	Female
13	Rest of Europe	> 16 years	Female
9	Rest of Europe	≤ 16 years	Male
9	Spain	≤ 16 years	Female
13	Rest of Europe	≤ 16 years	Female
9	Africa	> 16 years	Male
9	Spain	> 16 years	Female
13	Spain	≤ 16 years	Male
13	Rest of Europe	≤ 16 years	Male
13	Spain	> 16 years	Female
9	Africa	≤ 16 years	Male
9	Spain	> 16 years	Female
9	Africa	> 16 years	Male
13	Africa	≤ 16 years	Male
13	Spain	≤ 16 years	Female
9	Africa	≤ 16 years	Female
9	Spain	≤ 16 years	Male
9	Spain	≤ 16 years	Male
9	Spain	> 16 years	Male

13	Rest of Europe	≤ 16 years	Male

Table S3 Distribution of *Blastocystis* sp. alleles, country of origin of the patients, age and sex in sequences belonging to ST3

Allele	Geographical origin	Age	Sex
34	Rest of Europe	> 16 years	Female
34	Rest of Europe	≤ 16 years	Male
34	Africa	> 16 years	Female
34	Spain	≤ 16 years	Male
34	Rest of Europe	≤ 16 years	Female
34	Spain	> 16 years	Male
34	Spain	≤ 16 years	Male
36	Spain	> 16 years	Male
34	Spain	≤ 16 years	Female
34	Spain	> 16 years	Female
34	Africa	> 16 years	Female
34	Spain	> 16 years	Male
34	Spain	> 16 years	Female
34	Rest of Europe	≤ 16 years	Male
34	Africa	≤ 16 years	Female
34	Spain	> 16 years	Female
34	Africa	≤ 16 years	Female
34	Spain	≤ 16 years	Male
34	Asia	≤ 16 years	Male
34	Spain	≤ 16 years	Male
34	Spain	> 16 years	Female
34	Rest of Europe	≤ 16 years	Female
36	America	> 16 years	Female
34	Spain	> 16 years	Male
34	Spain	> 16 years	Female
36	Africa	≤ 16 years	Male
34	Spain	> 16 years	Female
34	Rest of Europe	≤ 16 years	Female
34	Africa	≤ 16 years	Male
34	Spain	≤ 16 years	Male
34	Spain	> 16 years	Male
34	Spain	≤ 16 years	Male

Table S4 Distribution of *Blastocystis* sp. alleles, country of origin of the patients, age and sex in sequences belonging to ST4

Allele	Geographical origin	Age	Sex
42	Spain	> 16 years	Male
42	Spain	≤ 16 years	Female
42	Spain	> 16 years	Male
42	Spain	> 16 years	Male
42	Spain	> 16 years	Male
42	Spain	≤ 16 years	Female
42	Spain	≤ 16 years	Female
42	Spain	≤ 16 years	Male
42	Spain	≤ 16 years	Male
42	Spain	≤ 16 years	Male
42	Spain	≤ 16 years	Male
42	Spain	> 16 years	Female
42	Spain	≤ 16 years	Female
42	Spain	> 16 years	Female
42	Spain	≤ 16 years	Male
42	Spain	≤ 16 years	Male

Table S5 **Comparisons between sequences of *Blastocystis* ST1 subtype found in this study and representative sequences deposited in GenBank**

Homology (%)	Nº secuence	Nº GenBank	Country	Origen
100.00	1008	MK244898.1	USA	*Bos taurus*
99.74	1008	AB107961.1	Japan	*Sus scrofa domesticus*
99.48	1008	MK874817.1	Mexico	*Homo sapiens*
97.15	1008	HQ641639.1	Spain	Primate - *Hapalemur aureus*
96.89	1008	OP020687.1	China	*Macaco Rhesus*
100.00	1137	MK801377.1	Germany	*Sus scrofa domesticus*
100.00	1137	GU992411.1	Philippines	Wastewater
99.47	1137	MK244899.1	USA	*Bos taurus*
99.47	1137	AB107967.1	Japan	*Pongo pygmaeus*
100.00	1173	MN326610.1	China	*Sus scrofa domesticus*
100.00	1173	HQ641595.1	Colombia	*Homo sapiens*
99.72	1173	MN918264.1	Poland	Agua de la laguna
99.72	1173	KU147338.1	Mexico	*Homo sapiens*
99.72	1173	KX358438.1	Laos	*Homo sapiens*
100.00	1246	ON834472.1	China	*Ailuropoda melanoleuca*
99.17	1246	OM057402.1	China	*Panthera leo*
98.33	1246	KX108707.1	Malaysia	*Homo sapiens*
96.39	1246	AB091240.1	Japan	*Gallus gallus domesticus*

Table S6 Comparisons between sequences of *Blastocystis* ST2 subtype found in this study and representative sequences deposited in GenBank

Homology (%)	Nº secuence	Nº GenBank	Country	Origen
100.00	1035	HQ641599.1	Colombia	*Homo sapiens*
100.00	1035	KF242003.1	Netherlands	*Homo sapiens*
100.00	1035	MW728061.1	Turkey	*Homo sapiens*
99.54	1035	OK599025.1	Spain	Aguas residuales reutilizadas
99.54	1035	MT186221.1	South Korea	*Homo sapiens*
100.00	1055	KY823332.1	China	*Macaca mulata*
100.00	1055	MW728087.1	Turkey	*Homo sapiens*
99.51	1055	ON287224.1	Iran	*Canis lupus familiaris*
99.52	1055	MG729833.1	Iran	*Homo sapiens*
100.00	1172	MN396290.1	Iran	*Homo sapiens*
99.70	1172	MW728082.1	Turkey	*Homo sapiens*
99.41	1172	KX524051.1	Brasil	*Homo sapiens*
99.11	1172	AM275383.1	Denmark	*Homo sapiens*
99.11	1172	MK874815.1	Mexico	*Homo sapiens*
99.11	1172	KX524009.1	Brasil	*Homo sapiens*
99.11	1172	MW728071.1	Turkey	*Homo sapiens*
99.11	1172	HQ641620.1	Colombia	*Homo sapiens*
98.52	1172	MK587496.1	Spain	*Vulpes vulpes*
98.52	1172	OM057419.1	China	*Equus zebra*
98.52	1172	HQ641645.1	Spain	*Cercopithecus hamlyni*
98.22	1172	MK801365.1	Germany	*Sus scrofa domesticus*
99.78	1198	MW728082.1	Turkey	*Homo sapiens*
99.34	1198	OM057419.1	China	*Equus zebra*
99.12	1198	ON834473.1	China	*Ailuropoda melanoleuca*
98.90	1198	MK587496.1	Spain	*Vulpes vulpes*
98.90	1198	HQ641645.1	Spain	*Cercopithecus hamlyni*
97.37	1198	KF306292.1	Libya	*Homo sapiens*
100.00	1255	MK801368.1	Germany	*Sus scrofa domesticus*
99.12	1255	KF242035.1	Netherlands	*Homo sapiens*
98.23	1255	HQ641617.1	Colombia	*Homo sapiens*
97.94	1255	MW728075.1	Turkey	*Homo sapiens*

Table S7 **Comparisons between sequences of *Blastocystis* ST3 subtype found in this study and representative sequences deposited in GenBank**

Homology (%)	Nº secuence	Nº GenBank	Country	Origen
100.00	1004	MZ700054.1	Mexico	*Homo sapiens*
100.00	1004	MZ664547.1	Spain	*Bos taurus*
99.77	1004	MN918263.1	Poland	Agua de río
99.77	1004	MT903362.1	South Korea	*Homo sapiens*
99.30	1004	HQ641610.1	Colombia	*Homo sapiens*
100.00	1156	EU445496.1	Philippines	*Homo sapiens*
99.76	1156	MN918265.1	Poland	Agua de lago
99.51	1156	MN264510.1	Iran	*Rattus norvegicus*
99.27	1156	HQ641610.1	Colombia	*Homo sapiens*
100.00	1135	KY290430.1	Malaysia	*Homo sapiens*
99.75	1135	KX234611.1	Malaysia	*Canis lupus familiaris*
95.35	1135	HQ641651.1	Spain	*Cercopithecus hamlyni*
94.65	1135	OM057426.1	China	*Macaca leonina*
93.95	1135	MN918265.1	Poland	Agua de lago
93.95	1135	MT903380.1	South Korea	*Homo sapiens*
93.95	1135	MW662481.1	Colombia	Uniq24-ST3
100.00	1369	MT089925.1	Egypt	*Homo sapiens*
99.78	1369	OM057422.1	China	*Erythrocebus patas*
98.89	1369	KF306291.1	Libya	*Homo sapiens*
98.89	1369	AB107969.1	Japan	*Macaca nemestrina*
97.78	1369	OM057419.1	China	*Equus zebra*
97.78	1369	MW728077.1	Turkey	*Homo sapiens*
100.00	1382	HQ641611.1	Colombia	*Homo sapiens*
99.75	1382	MN918261.1	Poland	Agua de río
99.51	1382	MN914081.1	Germany	*Homo sapiens*
98.77	1382	MK244903.1	USA	*Bos taurus*

Table S8 **Comparisons between sequences of *Blastocystis* ST4 subtype found in this study and representative sequences deposited in GenBank**

Homology (%)	Nº secuence	Nº GenBank	Country	Origen
100.00	1069	MK244908.1	USA	*Bos taurus*
99.75	1069	MT071885.1	China	Rata wistar
99.75	1069	AY590114.1	France	Rata wistar
99.75	1069	MK587493.1	Spain	*Vulpes vulpes*
99.75	1069	MT898452.1	USA	*Homo sapiens*
99.51	1069	ON394480.1	China	*Cygnus cygnus*
99.51	1069	MZ267667.1	USA	*Odocoileus virginianus*
99.51	1069	AY244619.1	Germany	*Homo sapiens*
99.01	1069	MH127500.1	Japan	*Rattus novercious*
98.77	1069	KX234618.1	Malaysia	*Canis lupus familiaris*
98.52	1069	OK235458.1	China	*Myocastor coypus*
97.95	1069	ON834467.1	China	*Ailuropoda melanoleuca*
100.00	1158	MK587493.1	Spain	*Vulpes vulpes*
100.00	1158	MK244907.1	USA	*Bos taurus*
100.00	1158	OM057428.1	China	*Erythrocebus patas*
100.00	1158	MZ267653.1	USA	*Odocoileus virginianus*
100.00	1158	AY590114.1	France	Rata wistar
99.32	1158	MW728074.1	Turkey	*Homo sapiens*
99.32	1158	MZ613336.1	China	*Moschus chrysogaster*
99.32	1158	MT114835.1	South Korea	*Hydropotes inermis argyropus*
99.32	1158	AB091251.1	Japan	*Rattus Norvegicus*
98.88	1158	MH127478.1	Indonesia	*Rattus exulans*
98.88	1158	KX234637.1	Malaysia	*Capra aegagrus hircus*
98.88	1158	MN264520.1	Iran	*Felis catus domesticus*
100.00	1288	MK587493.1	Spain	*Vulpes vulpes*
100.00	1288	MK244908.1	USA	*Bos taurus*
100.00	1288	OM865862.1	China	*Homo sapiens*
100.00	1288	OM057427.1	China	*Cavia porcellus*
100.00	1288	MT898453.1	Spain	*Homo sapiens*
100.00	1288	EU427516.1	Japan	*Dendrolagus goodfellowi*
99.53	1288	MW581486.1	Spain	*Ratus* spp.
99.53	1288	MW798739.1	China	*Homo sapiens*
99.53	1288	MH127487.1	Indonesia	*Rattus exulans*
100.00	1418	MK244908.1	USA	*Bos taurus*
100.00	1418	MK587493.1	Spain	*Vulpes vulpes*
100.00	1418	OM057428.1	China	*Erythrocebus patas*
99.78	1418	MZ267653.1	Spain	*Odocoileus virginianus*
99.56	1418	ON394480.1	China	*Cygnus cygnus*
99.12	1418	MH127494.1	Japan	*Rattus novercious*
98.69	1418	HQ641652.1	Spain	Primate - *Lemur catta*

Table S9 Nucleotide similarity among partial SSU-rRNA gene sequences identified as *Blastocystis* ST1 in this study

		ST1 sequences																
		**1008**	**1027**	**1064**	**1094**	**1095**	**1137**	**1167**	**1173**	**1185**	**1219**	**1242**	**1246**	**1310**	**1359**	**1365**	**1407**	**1424**
	**1008**																	
	**1027**	95.1																
	**1064**	95.2	99.4															
	**1094**	95.1	99.4	98.7														
	**1095**	95.6	99.4	100.0	98.9													
	**1137**	95.5	99.7	99.7	99.1	99.7												
	**1167**	95.5	99.4	100.0	98.9	100.0	99.7											
	**1173**	95.8	99.7	99.1	99.7	99.1	99.4	99.1										
**ST1 sequences**	**1185**	94.1	99.3	98.6	99.4	98.8	99.0	98.9	99.1									
	**1219**	95.9	99.7	99.5	99.1	99.5	99.7	99.5	99.4	99.1								
	**1242**	95.6	99.4	98.9	100.0	98.9	99.2	98.9	99.7	99.4	99.2							
	**1246**	96.1	95.3	96.0	94.4	96.1	95.8	96.1	94.9	94.2	95.6	95.1						
	**1310**	95.6	99.7	99.2	98.9	98.9	99.4	98.8	99.7	98.6	99.5	99.5	95.3					
	**1359**	95.6	99.7	99.2	99.4	99.3	99.4	99.2	99.7	99.1	99.5	99.5	95.3	100.0				
	**1365**	95.6	99.7	98.9	99.7	99.1	99.4	99.2	100.0	99.2	99.3	99.5	95.3	99.1	99.5			
	**1407**	95.9	99.7	99.2	99.7	99.3	99.4	99.2	100.0	99.2	99.5	99.8	95.3	99.4	99.8	99.8		
	**1424**	95.9	99.7	99.2	99.1	98.9	99.4	99.2	100.0	98.6	99.5	99.8	95.3	99.8	99.8	99.3	99.6	

Table S10 Nucleotide similarity among partial SSU-rRNA gene sequences identified as *Blastocystis* ST2 in this study

	ST2 sequences																														
	1028	1035	1036	1038	1055	1065	1068	1075	1081	1120	1124	1128	1133	1136	1152	1166	1172	1179	1195	1198	1215	1255	1311	1349	1354	1369	1381	1383	1392	1442	1447
	**99.08**																														
	**98.99**	100.0																													
	**98.98**	100.0	96.1																												
	**99.02**	100.0	100.0	100.0																											
	**98.62**	99.1	99.7	95.8	100.0																										
	**99.08**	100.0	96.1	99.5	100.0	96.2																									
	**98.84**	100.0	99.7	95.7	100.0	100.0	95.9																								
	**99.08**	100.0	98.9	95.7	100.0	98.2	96.2	98.5																							
	**99.08**	98.2	97.5	94.7	99.1	98.2	95.3	97.7	98.2																						
	**99.08**	100.0	96.1	99.5	100.0	96.3	100.0	96.0	96.2	95.5																					
	**99.08**	100.0	99.7	95.9	100.0	99.5	96.6	100.0	98.7	97.5	96.6																				
	**98.92**	100.0	99.7	95.8	100.0	100.0	96.1	100.0	98.5	97.8	96.1	100.0																			
	**99.54**	98.6	95.2	99.1	98.5	95.3	99.2	94.8	95.5	95.3	99.3	95.8	94.9																		
	**100.00**	98.9	97.5	94.7	98.9	98.1	95.1	97.7	98.5	100.0	95.3	97.7	97.8	95.1																	
ST2 sequences	**98.94**	100.0	99.7	95.7	100.0	100.0	96.3	100.0	98.5	97.9	96.3	100.0	100.0	95.1	97.9																
	**99.47**	99.5	99.4	95.6	99.5	99.7	95.6	99.7	98.2	97.9	95.6	99.7	99.7	95.3	97.9	99.7															
	**100.00**	98.9	95.4	98.9	98.9	95.7	99.5	95.4	95.1	95.7	99.5	95.3	95.6	99.7	95.7	95.7	95.6														
	**100.00**	99.1	97.5	94.7	99.1	98.1	95.3	97.7	98.6	100.0	95.5	97.8	97.8	95.3	100.0	97.9	97.9	95.7													
	**99.54**	98.6	98.9	94.9	98.5	98.9	95.8	99.1	97.9	98.1	96.1	99.3	99.2	96.1	98.2	99.2	99.4	95.9	98.3												
	**99.08**	100.0	99.7	95.8	100.0	99.5	96.8	100.0	98.7	97.7	96.8	100.0	100.0	95.8	97.9	100.0	99.7	95.7	97.9	99.3											
	**99.47**	98.4	99.1	95.1	98.4	98.8	95.1	98.8	97.9	97.6	95.2	98.8	98.8	95.3	97.6	98.8	99.1	95.6	97.6	99.1	98.8										
	**99.08**	100.0	100.0	95.8	100.0	99.3	96.8	99.7	98.9	97.5	96.8	99.8	99.7	95.8	97.6	99.7	99.4	95.7	97.7	99.1	99.8	99.1									
	**99.08**	100.0	96.1	99.5	100.0	96.5	99.6	96.0	96.2	95.6	100.0	96.5	96.2	99.6	95.6	96.7	95.6	99.5	95.7	96.3	96.8	95.1	96.8								
	**100.00**	99.1	95.5	98.9	99.1	95.9	99.5	96.0	95.7	95.8	99.5	96.1	95.6	99.8	95.8	95.7	95.6	100.0	95.9	96.6	96.3	95.6	96.3	99.5							
	**100.00**	98.9	97.4	94.8	98.9	97.7	94.8	97.6	98.5	100.0	94.8	97.6	97.7	94.9	100.0	97.7	97.9	95.4	100.0	97.9	97.7	97.6	97.4	94.8	95.4						
	**99.08**	100.0	96.1	100.0	100.0	96.1	100.0	95.8	96.2	95.2	100.0	96.5	96.1	99.2	95.1	96.1	95.6	99.4	95.2	95.9	96.6	95.1	96.6	100.0	99.5	94.8					
	**98.41**	99.5	99.4	95.4	99.5	99.7	95.6	99.7	98.2	97.7	95.9	99.7	99.7	94.8	97.7	99.7	99.4	95.5	97.7	98.9	99.7	99.1	99.5	96.1	95.5	97.4	95.7				
	**99.08**	100.0	96.1	100.0	100.0	96.1	100.0	95.8	96.2	95.1	100.0	96.5	96.1	99.2	95.1	96.1	95.6	99.4	95.2	95.8	96.6	95.1	96.6	100.0	99.5	94.8	100.0	95.8			
	**98.62**	99.5	99.7	95.5	99.5	99.1	96.5	99.4	98.7	97.2	96.5	99.5	99.4	95.9	97.4	99.5	99.1	95.5	97.5	98.8	99.5	98.8	99.7	96.5	96.1	97.1	96.3	99.2	96.3		
	**100.00**	99.1	97.5	94.7	99.1	97.8	95.6	97.7	98.7	99.6	95.8	97.9	97.8	95.7	100.0	97.9	97.9	95.7	100.0	98.4	98.1	97.6	97.9	96.1	96.3	100.0	95.6	97.7	95.6	97.6	

Table S11 Nucleotide similarity among partial SSU-rRNA gene sequences identified as *Blastocystis* ST3 in this study

		ST3 sequences																																									
		1001	1004	1010	1014	1015	1058	1072	1078	1079	1098	1099	1102	1106	1108	1135	1149	1156	1178	1186	1189	1193	1200	1202	1214	1234	1237	1254	1309	1313	1322	1347	1348	1356	1361	1364	1375	1380	1382	1384	1386	1428	1457	1459
	**1001**																																											
	**1004**	98.7																																										
	**1010**	98.7	99.8																																									
	**1014**	98.7	99.8	100.0																																								
	**1015**	100.0	98.8	98.8	98.8																																							
	**1058**	98.9	98.8	99.8	98.8	99.0																																						
	**1072**	98.9	98.8	99.8	98.8	99.0	100.0																																					
	**1078**	98.7	98.8	100.0	100.0	98.7	99.8	99.8																																				
	**1079**	98.9	98.8	99.8	99.8	99.0	100.0	100.0	99.8																																			
	**1098**	98.9	98.8	99.8	99.8	99.0	100.0	100.0	99.8	100.0																																		
	**1099**	98.6	98.8	99.8	99.8	98.8	99.8	99.8	99.8	99.8	99.8																																	
	**1102**	98.8	100.0	99.8	99.8	98.8	99.8	99.8	99.8	99.8	99.8	99.8																																
	**1106**	98.8	99.5	99.3	99.3	98.8	99.3	99.3	99.3	99.3	99.3	99.3	99.6																															
	**1108**	98.8	100.0	99.8	99.8	98.8	99.8	99.8	99.8	99.8	99.8	99.8	100.0	99.6																														
	**1135**	93.3	93.8	94.1	94.1	93.8	93.9	93.9	94.1	93.8	93.9	93.9	93.9	94.4	93.8																													
	**1149**	98.6	100.0	99.7	99.7	98.5	99.7	99.7	99.7	99.7	99.7	99.7	100.0	99.4	100.0	92.8																												
	**1156**	98.6	100.0	99.8	99.8	98.8	99.8	99.8	99.8	99.8	99.8	99.8	100.0	99.5	100.0	93.9	100.0																											
	**1178**	98.5	100.0	99.7	99.7	98.5	99.7	99.7	99.7	99.7	99.7	99.7	100.0	99.4	100.0	92.5	100.0	100.0																										
	**1186**	98.2	99.7	99.7	99.7	98.2	99.7	99.7	99.7	99.7	99.7	100.0	99.7	98.9	99.7	91.4	99.7	99.7	99.7																									
	**1189**	98.7	100.0	99.8	99.8	98.8	99.8	99.8	99.8	99.8	99.8	100.0	100.0	99.5	100.0	93.9	100.0	100.0	100.0	99.6																								
ST3 sequences	**1193**	99.3	99.53	99.3	99.2	99.3	99.3	99.3	99.3	99.3	99.3	99.3	99.6	99.6	99.6	93.9	99.4	99.5	99.4	98.9	99.5																							
	**1200**	98.8	100.0	99.8	99.8	98.8	99.8	99.8	99.8	99.8	99.8	99.8	100.0	99.6	100.0	93.8	100.0	100.0	100.0	99.7	100.0	99.6																						
	**1202**	99.1	99.8	99.8	99.8	99.0	100.0	100.0	99.8	100.0	100.0	99.8	99.8	99.3	99.8	93.8	99.7	99.8	99.7	99.7	99.8	99.3	99.8																					
	**1214**	99.1	99.8	99.8	99.8	98.9	100.0	100.0	99.8	100.0	100.0	99.8	99.8	99.3	99.8	93.8	99.7	99.8	99.7	99.7	99.8	99.4	99.8	100.0																				
	**1234**	98.8	100.0	99.8	99.8	98.8	99.8	99.8	99.8	99.8	99.8	99.8	100.0	99.6	100.0	93.8	100.0	100.0	100.0	99.7	99.8	99.6	100.0	99.8	99.8																			
	**1237**	98.8	100.0	99.8	99.8	98.8	99.8	99.8	99.8	99.8	99.8	99.8	100.0	99.6	100.0	93.8	100.0	100.0	100.0	99.7	100.0	99.6	100.0	99.8	99.8	100.0																		
	**1254**	98.8	99.7	99.5	99.5	98.9	99.5	99.5	99.5	99.5	99.5	99.5	99.7	99.7	99.7	93.9	99.7	99.7	99.7	99.3	99.7	99.7	99.7	99.5	98.9	99.7	99.7																	
	**1309**	98.9	99.8	99.8	99.8	99.0	100.0	100.0	99.8	100.0	100.0	99.8	99.8	99.3	99.8	93.9	99.7	99.8	99.7	99.7	99.8	99.3	99.8	100.0	100.0	99.8	99.8	99.5																
	**1313**	99.1	99.8	99.8	99.8	99.0	100.0	100.0	99.8	100.0	100.0	99.8	99.8	99.4	99.8	93.9	99.7	99.8	99.7	99.7	99.8	99.4	99.8	100.0	100.0	99.8	99.8	99.5	100.0															
	**1322**	98.5	100.0	99.7	99.8	98.5	99.7	99.7	99.8	99.7	99.7	99.7	100.0	99.4	100.0	92.6	100.0	100.0	100.0	99.7	100.0	99.4	100.0	99.7	99.7	99.7	100.0	99.7	99.7	99.7														
	**1347**	98.8	99.8	99.8	99.8	98.8	99.8	99.8	99.8	99.8	99.8	100.0	99.8	99.3	99.8	93.9	99.7	99.8	99.7	100.0	99.8	99.4	99.8	99.8	99.8	99.8	99.8	99.5	99.8	99.8	99.7													
	**1348**	99.1	99.8	99.8	99.8	99.0	100.0	100.0	99.8	100.0	100.0	99.8	99.8	99.3	99.8	93.9	99.7	99.8	99.7	99.7	99.8	99.4	99.8	100.0	100.0	99.8	99.8	99.5	100.0	100.0	99.7	99.8												
	**1356**	99.7	99.0	99.0	99.00	99.8	99.3	99.3	99.0	99.3	99.3	99.0	99.0	99.0	99.0	94.1	98.8	99.0	98.8	98.6	99.0	99.5	99.0	99.3	99.3	98.9	99.0	99.2	99.3	99.3	98.8	99.0	99.8											
	**1361**	98.8	99.8	99.8	99.8	98.8	99.8	99.8	99.8	99.8	99.8	100.0	99.8	99.3	99.8	93.9	99.7	99.8	99.7	100.0	99.8	99.4	99.8	99.8	99.8	99.8	99.8	99.5	99.8	99.8	99.7	100.0	99.8	99.0										
	**1364**	98.8	100.0	99.8	99.8	98.8	99.8	99.8	99.8	99.8	99.8	99.8	100.0	99.6	100.0	93.8	100.0	100.0	100.0	99.7	100.0	99.6	100.0	99.8	99.8	100.0	100.0	99.7	99.8	99.8	100.0	99.8	99.8	99.0	99.8									
	**1375**	98.8	100.0	99.8	99.8	98.8	99.8	99.8	99.8	99.8	99.8	99.8	100.0	99.6	100.0	93.8	100.0	100.0	100.0	99.7	100.0	99.6	100.0	99.8	99.8	100.0	100.0	99.7	99.8	99.8	100.0	99.8	99.8	98.9	99.8	100.0								
	**1380**	99.5	98.8	98.8	98.8	99.5	99.0	99.1	98.8	99.0	99.1	98.8	98.9	98.9	98.9	93.4	98.6	98.8	98.5	98.2	98.8	99.4	98.9	99.1	99.1	98.9	98.9	99.5	99.1	99.2	98.5	98.9	99.1	99.3	98.9	98.9	98.9							
	**1382**	99.7	98.8	99.0	99.0	99.8	98.7	98.8	99.0	98.7	98.8	98.7	98.8	98.8	98.8	94.0	98.6	98.7	98.5	98.2	98.8	99.3	98.8	98.8	98.8	98.8	98.8	98.9	98.8	98.8	98.5	98.8	98.8	99.5	98.8	98.8	98.8	99.3						
	**1384**	98.8	100.0	99.8	99.8	98.8	99.8	99.8	99.8	99.8	99.8	99.8	100.0	99.6	100.0	93.8	100.0	100.0	100.0	99.7	100.0	99.6	100.0	99.8	99.8	100.0	100.0	99.7	99.8	99.8	100.0	99.8	99.8	99.0	99.8	100.0	100.0	98.9	98.8					
	**1386**	99.1	99.8	99.8	99.8	98.9	100.0	100.0	99.8	100.0	100.0	99.8	99.8	99.3	99.8	93.8	99.7	99.8	99.7	99.7	99.8	99.3	99.8	100.0	100.0	99.8	99.8	99.5	100.0	100.0	99.7	99.8	100.0	99.2	99.8	99.8	99.8	99.1	98.8	99.8				
	**1428**	98.8	100.0	99.8	99.8	98.8	99.8	99.8	99.8	99.8	99.8	99.8	100.0	99.6	100.0	93.8	100.0	100.0	100.0	99.7	100.0	99.6	100.0	99.8	99.8	100.0	100.0	99.7	99.8	99.8	100.0	99.8	99.8	99.0	99.8	100.0	100.0	98.9	98.8	100.0	99.8			
	**1457**	99.8	98.8	99.2	99.8	99.8	98.8	98.8	99.8	98.8	98.8	98.8	98.8	98.8	98.8	94.1	98.6	98.8	98.5	98.2	98.8	99.3	98.8	98.8	98.8	100.0	98.8	98.9	98.8	98.8	98.5	98.8	98.8	99.5	98.8	98.8	98.8	99.3	100.0	98.8	98.8	98.8		
	**1459**	98.7	99.8	99.8	99.8	98.8	99.8	99.8	99.8	99.8	99.8	100.0	99.8	99.3	99.8	93.8	99.7	99.8	99.7	100.0	99.8	99.3	99.8	99.8	99.8	99.8	99.8	99.5	99.8	99.8	99.7	100.0	99.8	99.0	100.0	99.8	99.8	98.8	98.8	99.8	99.8	99.8	98.8	

Table S12 Nucleotide similarity among partial SSU-rRNA gene sequences identified as *Blastocystis* ST4 in this study

		ST4 sequences																			
		**1069**	**1074**	**1082**	**1123**	**1148**	**1158**	**1181**	**1192**	**1205**	**1211**	**1225**	**1250**	**1284**	**1288**	**1315**	**1316**	**1393**	**1398**	**1418**	**1419**
	**1069**																				
	**1074**	99.7																			
	**1082**	99.7	100.0																		
	**1123**	99.8	100.0	100.0																	
	**1148**	99.7	100.0	100.0	100.0																
	**1158**	99.8	100.0	100.0	100.0	100.0															
	**1181**	99.8	100.0	100.0	100.0	100.0	100.0														
	**1192**	99.8	100.0	100.0	100.0	100.0	100.0	100.0													
	**1205**	99.8	100.0	100.0	100.0	100.0	100.0	100.0	100.0												
**ST4 sequences**	**1211**	99.8	100.0	100.0	100.0	100.0	100.0	100.0	100.0	100.0											
	**1225**	99.8	100.0	100.0	100.0	100.0	100.0	100.0	100.0	100.0	100.0										
	**1250**	99.8	100.0	100.0	100.0	100.0	100.0	100.0	100.0	100.0	100.0	100.0									
	**1284**	99.8	100.0	100.0	100.0	100.0	100.0	100.0	100.0	100.0	100.0	100.0	100.0								
	**1288**	100.0	100.0	100.0	100.0	100.0	100.0	100.0	100.0	100.0	100.0	100.0	100.0	100.0							
	**1315**	99.8	100.0	100.0	100.0	100.0	100.0	100.0	100.0	100.0	100.0	100.0	100.0	100.0	100.0						
	**1316**	99.8	100.0	100.0	100.0	100.0	100.0	100.0	100.0	100.0	100.0	100.0	100.0	100.0	100.0	100.0					
	**1393**	99.8	100.0	100.0	100.0	100.0	100.0	100.0	100.0	100.0	100.0	100.0	100.0	100.0	100.0	100.0	100.0				
	**1398**	99.8	100.0	100.0	100.0	100.0	100.0	100.0	100.0	100.0	100.0	100.0	100.0	100.0	100.0	100.0	100.0	100.0			
	**1418**	100.0	99.8	99.7	99.8	99.7	99.8	99.8	99.8	99.8	99.8	99.8	99.8	99.8	100.0	99.8	99.8	99.8	99.8		
	**1419**	99.8	100.0	100.0	100.0	100.0	100.0	100.0	100.0	100.0	100.0	100.0	100.0	100.0	100.0	100.0	100.0	100.0	100.0	99.8	

Table S13 Distribution of *Blastocystis* sp. subtypes by continents

Continent	Country origin of sample	Diagnostic technique	Total	ST1 (%)	ST2 (%)	ST3 (%)	ST4 (%)	ST5 (%)	ST6 (%)	ST7 (%)	ST8 (%)	ST12 (%)	ST16 (%)	ST Mixed (%)	ST Unknown/ ST new (%)	Reference
America																
	USA	SEC	9	1 (11)	-	6 (67)	-	-	-	-	-	-	-	-	2 (22)	Jones et al, 2009
	Colombia	SEC	14	4 (28,6)	1 (7,1)	3 (21,4)	2 (14,3)	-	-	-	-	-	-	4 (28,6)	-	Santin et al, 2011
	Brasil	SEC	80	33 (41)	27 (34)	13 (16)	-	-	-	-	-	-	-	7 (9)	-	Malheiros et al, 2011
	USA	SEC	10	2 (20)	3 (30)	5 (50)	-	-	-	-	-	-	-	-	-	Scanlan et al, 2016
	Bolivia	SEC	40	-	13 (32,5)	12 (30)	-	8 (20)	-	-	-	3 (7,5)	-	-	4 (10)	
	Ecuador	SEC	25	3 (12)	-	21 (84)	-	-	-	-	-	-	-	-	1 (4)	
	Peru	SEC	13	-	-	12 (92)	-	-	-	-	-	-	-	-	1 (8)	Ramírez et al, 2016
	Brasil	SEC	22	2 (9)	7 (32,5)	8 (36)	-	-	-	1 (4,5)	2 (9)	-	-	-	2 (9)	
	Colombia	SEC	181	82 (45)	47 (26)	33 (18)	7 (4)	-	4 (2)	7 (4)	-	-	-	-	1 (1)	
	Argentina	SEC	65	11 (17)	10 (15)	41 (63)	-	-	3 (5)	-	-	-	-	-	-	
	Brasil	STS	64	23 (36)	4 (6)	27 (42)	1 (2)	-	-	-	-	-	-	-	9 (14)	Valença Barbosa et al, 2017
	Colombia	SEC	256	52 (20,31)	47 (18,4)	134 (52,34)	2 (0,8)	-	5 (1,9)	2 (0,8)	-	-	-	-	14 (5,5)	Ramírez et al, 2017
	Brasil	SEC	57	31 (54,4)	4 (7)	19 (33,3)	-	-	-	3 (5,3)	-	-	-	-	-	Oliveira-Arbex et al, 2018
	Colombia	SEC	59	12 (20,3)	14 (23,7)	18 (30,5)	3 (5,1)	-	1 (1,7)	-	-	-	9 (15,3)	2 (3,4)	-	Osorio-Pulgarin 2021

Continent	Country origin of sample	Diagnostic technique	Total	ST1 (%)	ST2 (%)	ST3 (%)	ST4 (%)	ST5 (%)	ST6 (%)	ST7 (%)	ST8 (%)	ST9 (%)	ST Mixed (%)	ST U/ ST n (%)		Reference
Europe																
	Germany	RFLP	78	18 (23)	1 (1,3)	51 (65,4)	5 (6,4)	-	-	-	-	-	-	3 (3,8)	-	Bohm-Gloning et al, 1997
	UK	RFLP	29	2 (6,9)	1 (3,4)	22(75,9)	4 (13,8)	-	-	-	-	-	-	-	-	Clark 1997
	Germany	STS	12	3 (25)	2 (16,7)	5 (41,7)	2 (16,7)	-	-	-	-	-	-	-	-	Yoshikawa et al, 2004
	Denmark	SEC	29	1 (3,4)	6 (20,7)	15 (51,7)	7 (24,1)	-	-	-	-	-	-	-	-	Stensvold et al, 2006
	UK	SEC	49	2 (4)	8 (16,3)	20 (40,8)	16(32,7)	-	-	1 (2)	1 (2)	-	1 (2)	-	-	Scicluna et al, 2006
	Denmark	SEC	28	5 (17,9)	9 (32,1)	13 (46,4)	1 (3,6)	-	-	-	-	-	-	-	-	Stensvold et al, 2007
	Greece	SSCP	45	9 (20)	6 (13,3)	27 (60)	1 (2,2)	-	1 (2,2)	1 (2,2)	-	-	-	-	-	Menounos et al, 2008
	Ireland	SEC	14	1 (7)	6 (43)	4 (29)	3 (21)	-	-	-	-	-	-	-	-	Scanlan and Marchesi 2008
	Spain	RFLP	51	1 (1,9)	2 (3,9)	-	48 (94,2)	-	-	-	-	-	-	-	-	Domínguez-Márquez et al, 2009
	France	SEC	46	11 (24)	4 (8,5)	23 (50)	4 (8,5)	-	-	1 (2)	-	-	3 (7)	-	-	Souppart et al, 2009
	Denmark	SEC	24	2 (8,3)	3 (12,5)	6(25)	9 (37,5)	-	-	-	-	1 (4,2)	3 (12,5)	-	-	Stensvold et al, 2009
	Denmark	SEC	99	20 (20,2)	15 (15,2)	39 (39,4)	16 (16,1)	-	1 (1)	-	1 (1)	-	7 (7)	-	-	Rene et al, 2009
	Denmark	SEC	25	1 (4)	4 (16)	1 (4)	19 (76)	-	-	-	-	-	-	-	-	Stensvold et al, 2011a
	Denmark	SEC	22	9 (41)	-	11 (50)	-	-	-	-	-	-	2 (9)	-	-	Stensvold et al, 2011b
	France	SEC	27	1 (3,7)	1 (3,7)	4 (14,8)	17 (63)	-	1 (3,7)	3 (11,1)	-	-	-	-	-	Poirier et al, 2011
	Italy	SEC	38	3 (8)	7 (18)	16 (42)	6 (16)	-	-	1 (2,5)	1 (2,5)	-	4 (11)	-	-	Meloni et al, 2011
	Sweden	SEC	63	10 (15,9)	9 (14,3)	30 (47,6)	13 (20,6)	-	-	1 (1,6)	-	-	-	-	-	Forsell et al, 2012
	Netherlands	SEC	103	23 (22)	24 (22)	43 (42)	12 (12)	-	-	-	-	-	1 (1)	-	-	Bart et al, 2013
	UK	SEC	271	34 (13)	26 (10)	114 (42)	85 (31)	2 (0,7)	1 (0,3)	4 (1)	5 (2)	-	-	-	-	Alfellani et al, 2013
	France	SEC	143	28 (20)	18 (12)	61 (43)	28 (20)	-	3 (2)	3 (2)	-	-	2 (1)	-	-	El Safadi et al, 2016
	Spain	SEC	53	7 (13,2)	33 (62,3)	9 (17)	4 (7,5)	-	-	-	-	-		-	-	Paulos et al., 2018
	Poland	STS	122	16 (13,1)	24 (19,7)	72 (59)	-	-	4 (3,3)	4 (3,3)	-	-	2 (1,6)	-	-	Rudzińska et al, 2019
	Spain	SEC	162	37 (23)	59 (36)	35 (22)	30 (19)	-	-	-	1 (1)	-	-	-	-	Muadica et al, 2020
	Italy	NGS	142	41 (29)	23 (16)	57 (40)	12 (17)	-	-	3 (4)	-	-	-	-	-	Gabrielli et al, 2020
	Spain	SEC	26	1 (3,8)	12 (46,2)	4 (15,4)	9 (34,6)	-	-	-	-	-	-	-	-	Hernández-Castro et al, 2023

Continent	Country origin of sample	Diagnostic technique	Total	ST1 (%)	ST2 (%)	ST3 (%)	ST4 (%)	ST6 (%)	ST7 (%)	ST10 (%)	ST14 (%)	ST Mixed (%)	ST Unknown/ ST new (%)			Reference
Africa																
	Egypt	STS	44	8 (18,2)	-	24 (54,5)	-	8 (18,2)	4(9,1)	-	-	-	-	-	-	Hussein et al., 2008
	Egypt	SEC	20	3(15)	4 (20)	12 (60)	-	-	-	-	-	1 (5)	-	-	-	Souppart et al., 2010
	Egypt	STS	120	15 (12,5)	-	49 (41)	-	33 (27,5)	13 (10,8)	-	-	10 (8,2)	-	-	-	Fouad et al., 2011
	Liberia	SEC	30	7 (23,4)	7 (23,4)	8 (26,6)	3 (10)	-	-	-	-	5 (16,6)	-	-	-	Alfellani et al, 2013
	Libya	SEC	38	19 (50)	3 (8)	15 (39,4)	-	-	1 (2,6)	-	-	-	-	-	-	Alfellani et al, 2013
	Nigeria	SEC	23	10 (43,5)	-	9 (39,2)	3 (13)	-	-	-	-	1 (4,3)	-	-	-	Alfellani et al, 2013
	Senegal	SEC	93	25 (27)	15 (16)	45 (48)	-	-	-	-	-	8 (9)	-	-	-	El Safadi et al, 2014
	Tunisia	SEC	61	18 (30)	10 (16)	31 (51)	1 (1,6)	-	1 (1,6)	-	-	-	-	-	-	Abda et al, 2017
	Senegal	SEC	588	113 (19)	226 (38)	107 (18)	-	-	3 (0,5)	2 (0,3)	2 (0,3)	135 (23)	-	-	-	Khaled et al, 2020
	Guinea	SEC	390	96 (24,5)	66 (16,9)	117 (30)	2 (0,5)	-	-	-	10 (3)	-	99 (25)	-	-	Guilavogui et al, 2022

Table S13 Distribution of *Blastocystis* sp. subtypes by continents

Continent	Country origin of sample	Diagnostic technique	Total	ST1 (%)	ST2 (%)	ST3 (%)	ST4 (%)	ST5 (%)	ST6 (%)	ST7 (%)	ST10 (%)	ST Mixed (%)	ST U/ ST n (%)	Reference
Central Asia-ME														
	Bangladesh	STS	26	2 (7,7)	-	24 (92)	-	-	-	-	-	-	-	Yoshikawa et al, 2004
	Pakistan	STS	10	2 (20)	-	7(70)	-	-	1 (10)	-	-	-	-	Yoshikawa et al, 2004
	Iran	RFLP	45	20 (44,4)	4 (8,9)	16 (35,6)	-	-	2 (4,4)	3 (6,7)	-	-	-	Motazedian et al, 2008
	Turkey	SEC	87	8 (9,2)	12 (13,8)	66 (75,9)	1 (1,2)	-	-	-	-	-	-	Özyurt et al, 2008
	Turkey	STS	92	17 (18,5)	20 (21,7)	51 (55,4)	-	-	-	-	-	4 (4,3)	-	Dogruman-Al et al, 2008
	Turkey	STS	32	20 (63)	3 (9)	9 (28)	-	-	-	-	-	-	-	Eroglu et al, 2009
	Nepal	STS	20	4 (20)	4 (20)	12 (60)	-	-	-	-	-	-	-	Yoshikawa et al, 2009
	Turkey	STS	35	1 (2,9)	10 (28,6)	21 (60)	-	-	-	-	-	3 (8,6)	-	Dogruman-Al et al, 2009 a
	Turkey	STS	66	10 (15)	9 (14)	38 (58)	-	-		-	-	9 (13)	-	Dogruman-Al et al, 2009 b
	Pakistan	STS	179	87 (49)	10 (5,5)	49 (27,3)	8 (4,4)	7 (3,9)	6 (3,3)	10 (5,5)	-	-	2 (1)	Yakoob et al, 2010
	Turkey	STS	25	9 (36)	6 (24)	10 (40)	-	-	-	-	-	-	-	Eroglu and Koltas 2010
	Iran	STS	174	48 (28)	7 (4)	53 (30)	-	33 (19)	-	-	-	33 (19)	-	Moosavi et al, 2012
	Iran	STS	150	40 (27)	-	-	-		8 (5,3)	53 (35,3)	-	41 (27)	8 (5,3)	Lee et al, 2012
	Lebanon	SEC	36	11 (30,6)	12 (33,3)	12 (33,3)	1 (2,8)	-	-	-	-	-	-	El Safadi et al, 2013
	Qatar	SEC	114	31 (27,2)	4 (3,5)	79 (69,3)	-	-	-	-	-	-	-	Abu-Madi et al, 2015
	UAE	SEC	39	11 (28,2)	3 (7,6)	23 (58,9)	-	-	-	-	-	2 (5,1)	-	AbuOdeh et al, 2016
	Saudi Arabia	STS	50	19 (38)	20 (40)	-	-	11 (22)	-	-	-	-	-	Mohamed et al, 2017 a
	Saudi Arabia	SEC	133	19 (14,5)	7 (5)	107 (80,5)	-	-	-	-	-	-	-	Mohamed et al, 2017 b
	Iran	SEC	41	8 (19,5)	8 (19,5)	25 (61)	-	-	-	-	-	-	-	Mardani Kataki et al, 2019
	Siria	SEC	195	43 (22)	31 (15,8)	89 (45,7)	-	-	-	-	1 (0,5)	31 (16)	-	Khaled 2021
	Turkey	SEC	15	3 (20)	5 (33)	6 (40)	-	-	-	-	-	1 (6)	-	Mülayim 2011
	Saudi Arabia	SEC	96	51 (53,13)	44 (45,85)	1 (1,04)	-	-	-	-	-	-	-	Wakid et al, 2022
	Iran	SEC	13	13 (39,4)	5 (15,2)	14 (42,4)	-	-	-	1 (3)	-	-	-	Rahimi et al, 2022

Continent	Country origin of sample	Diagnostic technique	Total	ST1 (%)	ST2 (%)	ST3 (%)	ST4 (%)	ST6 (%)	ST7 (%)	ST9 (%)	ST Mixed (%)	ST U/ ST n (%)		Reference
E-SE Asia														
	Japan	STS	32	1 (3,1)	-	30 (93,8)	-	1 (3,1)	-	-	-	-	-	Yoshikawa et al, 2000
	Japan	RFLP	55	11 (20)	12 (21,8)	24 (46,6)	6 (10,9)	2 (3,6)	-	-	-	-	-	Kaneda et al, 2001
	Thailand	RFLP	153	7 (4,6)	-	138 (90,2)	-	2 (1,3)	-	-	6(3,9)	-	-	Thathaisong et al, 2003
	Japan	STS	50	4(8)	-	26 (52)	2(4)	11 (22)	5 (10)	2(4)	-	-	-	Yoshikawa et al, 2004
	Thailand	STS	4	1 (25)	-	1 (25)	-	1 (25)	-	-	1 (25)	-	-	Yoshikawa et al, 2004
	Philippines	RFLP	12	10 (83)	-		-	-	-	-	-	2 (17)	-	Rivera and Tan 2005
	China	STS	35	13 (37,1)	2 (5,7)	14 (40)	-	-	-	-	5 (14,3)	1 (2,9)	-	Yan et al, 2006
	China	STS	192	47 (24,5)	9 (4,7)	116 (60,4)	1 (0,5)	1 (0,5)	-	-	10 (5,2)	8 (4,1)	-	Li et al, 2007 a
	China	STS	78	16 (20,5)	1 (1,3)	55 (70,5)	1 (1,3)	-	-	-	2(2,5)	3 (3,9)	-	Li et al, 2007 b
	Philippines	SEC	12	4 (33,3)	2 (16,7)	5 (41,7)	-	1 (8,3)	-	-	-	-	-	Rivera et al, 2008
	Singapur	RFLP	9	2 (22)	-	7 (78)	-	-	-	-	-	-	-	Wong et al, 2008
	Malaysia	STS	20	9 (45)	1 (5)	10 (50)	-	-	-	-	-	-	-	Tan et al, 2008
	Malaysia	STS	40	5 (12,5)	-	20 (50)	-	11 (27,5)	2 (5)	-	-	2 (5)	-	Tan et al, 2009
	Thailand	SEC	77	24 (31)	11 (14)	41 (53)	1 (2)	-	-	-	-	-	-	Popruk et al, 2015
	Tanzania	SEC	106	36 (34)	28 (26,4)	27 (25,5)	-	-	1 (0,9)	-	-	14 (13,2)	-	Forsell et al, 2016
	India	SEC	65	6 (9)	-	59 (91)	-	-	-	-	-	-	-	Das et al, 2016
	Philippines	SEC	29	9 (31,03)	-	19 (65,5)	1 (3,44)	-	-	-	-	-	-	Adao et al, 2016
	China	SEC	27	12 (44)	-	15 (56)	-	-	-	-	-	-	-	Zhang et al, 2017
	Malaysia	SEC	191	63 (33)	27 (14)	98 (51)	3 (2)	-	-	-	-	-	-	Noradilah et al, 2017
	Thailand	SEC	41	7 (17)	1 (2)	28 (68)	1 (2)	1 (2)	3 (7)	-	-	-	-	Yowang et al, 2018
	China	SEC	22	7 (32)	-	13 (59)	2 (9)	-	-	-	-	-	-	Gong et al, 2019
	Thailand	RFLP	109	34 (31,2)	11 (10,1)	64 (58,72)	-	-	-	-	-	-	-	Srichaipon et al, 2019
	Indonesia	SEC	43	20 (47)	2 (4)	21 (49)	-	-	-	-	-	-	-	Kesuma et al, 2019

Continent	Country origin of sample	Diagnostic technique	Total	ST1 (%)	ST2 (%)	ST3 (%)	ST4 (%)	ST6 (%)	ST7 (%)	ST8 (%)	ST Mixed (%)			Reference
Australia														
	Australia	SEC	13	5 (38)	-	4 (31)	2 (15)	1 (8)	-	-	1 (8)	-	-	Nagel et al, 2012
	Australia	SEC	91	28 (31)	5 (6)	40 (44)	12 (13)	3 (3)	1 (1)	2 (2)	-	-	-	Roberts et al, 2013

Table S14 Frequency of subtypes (%) of *Blastocystis* sp. identified in human infections in various studies in Spain

Subtypes	Valencia[a]	Álava[b]	Leganés (Madrid)[c]	Majadahonda (Madrid)[d]	Zaragoza[e]
	Frequency (%)	Frequency (%)	Frequency (%)	Frequency (%)	Frequency (%)
**ST1**	1 (1,9)	7 (13,2)	37 (22,8)	1 (3,8)	27 (15,61)
**ST2**	2 (3,9)	33 (62,3)	59 (36,4)	12 (46,2)	59 (34,1)
**ST3**		9 (17)	35 (21,6)	4 (15,4)	60 (34,68)
**ST4**	48 (94,2)	4 (7,5)	30 (18,5)	9 (34,6)	27 (15,61)
**ST8**	-	-	1 (0,7)	-	-
	**51 (100)**	**53 (100)**	**162 (100)**	**26 (100)**	**173 (100)**

[a] (Domínguez-Márquez et al., 2009); [b] Paulos et al., (2018); [c] Muadica et al., (2020); [d] (Hernández-Castro et al., 2023); [e]Present study
